# Endoplasmic Reticulum Stress–Induced Resistance to Doxorubicin Is Reversed by Paeonol Treatment in Human Hepatocellular Carcinoma Cells

**DOI:** 10.1371/journal.pone.0062627

**Published:** 2013-05-03

**Authors:** Lulu Fan, Bing Song, Guoping Sun, Tai Ma, Fei Zhong, Wei Wei

**Affiliations:** 1 Department of Oncology, The First Affiliated Hospital of Anhui Medical University, Hefei, Anhui, China; 2 Department of Cardiovasology, The First Affiliated Hospital of Anhui Medical University, Hefei, Anhui, China; 3 Institute of Clinical Pharmacology, Anhui Medical University, Hefei, Anhui, China; Complutense University, Spain

## Abstract

**Background:**

Endoplasmic reticulum stress (ER stress) is generally activated in solid tumors and results in tumor cell anti-apoptosis and drug resistance. Paeonol (Pae, 2-hydroxy-4-methoxyacetophenone), is a natural product extracted from the root of Paeonia Suffruticosa Andrew. Although Pae displays anti-neoplastic activity and increases the efficacy of chemotherapeutic drugs in various cell lines and in animal models, studies related to the effect of Pae on ER stress–induced resistance to chemotherapeutic agents in hepatocellular carcinoma (HCC) are poorly understood.

**Methodology/Principal Findings:**

In this study, we investigated the effect of the endoplasmic reticulum (ER) stress response during resistance of human hepatocellular carcinoma cells to doxorubicin. Treatment with the ER stress-inducer tunicamycin (TM) before the addition of doxorubicin reduced the rate of apoptosis induced by doxorubicin. Interestingly, co-pretreatment with tunicamycin and Pae significantly increased apoptosis induced by doxorubicin. Furthermore, induction of ER stress resulted in increasing expression of COX-2 concomitant with inactivation of Akt and up-regulation of the pro-apoptotic transcription factor CHOP (GADD153) in HepG2 cells. These cellular changes in gene expression and Akt activation may be an important resistance mechanism against doxorubicin in hepatocellular carcinoma cells undergoing ER stress. However, co-pretreatment with tunicamycin and Pae decreased the expression of COX-2 and levels of activation of Akt as well as increasing the levels of CHOP in HCC cells.

**Conclusions/Significance:**

Our results demonstrate that Pae reverses ER stress–induced resistance to doxorubicin in human hepatocellular carcinoma cells by targeting COX-2 mediated inactivation of PI3K/AKT/CHOP.

## Introduction

Hepatocellular carcinoma (HCC) is one of the most common solid malignancies characterized by a high prevalence of drug resistance. The development of drug resistance to chemotherapeutic agents in HCC cells is the major cause of chemotherapy failure in HCC patients [1]–[2]. The molecular mechanisms that underlie chemotherapeutic resistance are poorly understood.

A number of cellular stress conditions, such as nutrient deprivation, hypoxia, and alterations in glycosylation status, lead to the accumulation of unfolded and/or misfolded proteins in the lumen of the endoplasmic reticulum that is termed ER stress [3]–[4]. The ER responds to stress conditions by activating a range of signaling pathways that couples the ER protein folding load with the ER protein folding capacity and is termed the unfolded protein response (UPR) [5]–[6]. The activation of the UPR is believed to alleviate ER stress and promote cell survival via the activation of survival or proliferative pathways [7]–[9]. Hypoxia and anoxia are pathophysiologic characteristics of most solid tumors [10]–[11]. Evidence is emerging that hypoxia and anoxia play an important role in drug resistance of solid tumors [12]–[13], but how these processes contribute to chemoresistance in HCC cells remains to be explored. Both hypoxia and anoxia are known to cause ER stress and initiate the UPR [14]. Therefore, ER stress from tumor hypoxia may lead to drug resistance.

Paeonol (Pae), a major active component extracted from the herb Pycnostelma paniculatum (Bunge) K Schum and the root cortex of Paeonia suffruticosa Andrews, possesses extensive pharmacological activities such as sedation, hypnosis, antipyresis, analgesic, anti-oxidation, anti-inflammation, and immunoregulation [15]–[16]. Moreover, Pae displays oncostatic actions and increases the efficacy of chemotherapeutic drugs [17]–[19]. However, studies related to the effect of Pae on ER stress–induced resistance to chemotherapeutic agents in HCC are limited. In this study, we examined how the induction of ER stress results in increased resistance of hepatocellular carcinoma cells to apoptosis induced by chemotherapeutic drugs. Furthermore, we provide evidence that Pae reverses the effects of ER stress–induced resistance to doxorubicin by a COX-2 mediated inactivation of the PI3K/AKT/CHOP pathway.

## Materials and Methods

### Reagents

Tunicamycin (TM), 3-(4,5-dimethylthiazol-2-yl)-2,5 diphenyltetrazolium bromide (MTT), the PI3K inhibitor LY294002, RNase A and propidium iodide (PI) were obtained from Sigma Chemical (St. Louis, MO, USA). The Pae injection was purchased from First Pharmaceutical Factory of Shanghai (Shanghai, China). Doxorubicin was purchased from Shenzhen Main Luck Pharmaceuticals Inc. (Shenzhen, China). The COX-2 inhibitor, celecoxib, was obtained from Pfizer Corporation (New York, NY,USA). DMEM was obtained from Gibco BRL Life Technologies (Grand Island, NY, USA). The anti-GRP78, anti-CHOP, anti-β-actin antibodies were purchased from Santa Cruz Biotechnology (Santa Cruz, CA, USA). The anti-AKT and anti-COX-2 antibody was purchased from Abcam (Cambridge, MA, USA). The phospho (p)-Akt (Ser473) antibody and anti-Caspase-3 antibody was obtained from Cell Signaling Technology, Inc. (Danvers, MA, USA). Terminal deoxynucleotidyl transferase-mediated dUTP-biotin nick end labeling (TUNEL) System was purchased from Roche (Indianapolis, IN, USA). Trizol reagent and SYBR Green PCR master mix kit was obtained from Invitrogen (California, USA). RevertAid Premium First Strand cDNA Synthesis Kit was from Fermentas (Burlington, Canada).

### Cell Culture

Human hepatoma cell line (HepG2) was purchased from Shanghai cell bank (Chinese Academy of Sciences, Shanghai, China) and cultured in DMEM. HepG2 cells were supplemented with 10% (v/v) heat-inactivated fetal bovine serum (FBS), 100 unit/ml of penicillin and 100 µg/ml of streptomycin. Cultures were maintained in a humidified incubator at 37°C in 5% CO_2_.

### MTT Assay

Cells were cultured at a density of 1×10^4^ cells/well in a 96-well plate. After treatment with various concentrations of drug for the indicated time periods, MTT solution (5.0 mg/mL in phosphate buffered saline) was added (20.0 µL/well), and the plates were incubated for another 4 h at 37°C. The purple formazan crystals were dissolved in 150 µl of dimethyl sulfoxide (DMSO) per well. After 10 min, the plates were read on ELX800 universal microplate reader (Bio-Tek Instruments Inc, Winosski, VT) at 490 nm. Viability assays were performed by three independent experiments.

### Flow Cytometry

Cells were grown in 6-well plates and then treated with desired concentrations of the indicated compounds. After exposure to the indicated compounds for specific time periods, cells were trypsinized, washed twice with cold PBS and centrifuged. The cell pellet was resuspended in 1 mL of cold PBS and fixed in 9 mL of 70% ethanol at −20°C for at least 12 h. Then cells were centrifuged and resuspended in 500 µL PBS, RNase A was added and incubated at 37°C for 30 min. Propidium iodide (PI) staining buffer was added in the dark at room temperature for 30 min. A minimum of 1×10^6^ cells/mL for each group was analyzed using an EPICS XL-MCL model counter (Beckman Coulter, Fullerton, CA, USA).

### TUNEL Assay

Cells were cultured in 6-well plates on coverslips overnight. After treatment with various concentrations of the indicated compounds for each time period, the coverslips were washed twice with cold PBS and fixed in 4% paraformaldehyde solution for 1 h at room temperature. Apoptotic cells were detected by the TUNEL assay (TUNEL System Kit, Roche), which was performed according to the manufacturer’s instructions. The TUNEL assay results were quantitatively analyzed through the biological image analysis system from the Nikon ECLIPSE 80i biology microscope and Nikon Digital Camera DXM 1200F, ACT-1 version 2.63 software (Japan).

### Western Blotting

After drug treatment for the indicated time periods and concentrations, cells were lysed in RIPA lysis buffer (50 mmol/L TRIS (tris (hydroxymethyl) aminomethane)-HCl, (pH 7.4), 150 mmol/L NaCl, 10 mmol/L phenylmethylsulfonyl fluoride (PMSF), 1 mmol/L ethylene diamine tetraacetic acid (EDTA), 0.1% sodium dodecyl sulfate (SDS), 1% Triton X-100 and 1% sodium deoxycholate for 20–30 min on ice. Protein concentrations were determined through the Lowry Protein Assay. Lysates were incubated with 2X Laemmli sample buffer (Bio-Rad, Hercules, CA) and heated for 10 min at 95°C. Proteins were resolved by sodium dodecyl-sulfate-polyacrylamide gel electrophoresis (SDS-PAGE), transferred to polyvinylidene fluoride (PVDF) membranes (Millipore, Bedford, MA), and incubated with blocking buffer (Tris-buffered saline/Tween 20 (TBST) in 5% nonfat dry milk) overnight at 4°C. Immunoblots were incubated with the indicated primary antibody followed by the appropriate horseradish peroxidase (HRP)-conjugated secondary antibody, and visualized with enhanced chemiluminescence (ECL, Pierce, Rockford, IL) using hydrogen peroxide and luminol as substrate with Kodak X-AR film. Autoradiographs were scanned using a GS-700 Imaging Densitometer (Bio-Rad, Hercules, CA).

### Quantitative Real-time PCR (qRT-PCR)

Total RNA was extracted from HepG2 cells using Trizol reagent (Invitrogen, California, USA), 1 µg RNA was reverse-transcribed to cDNA using RevertAid Premium First Strand cDNA Synthesis Kit (Fermentas, Burlington, Canada). To determine the quantity of mRNA, the cDNA was amplified by real-time PCR with SYBR Green PCR master mix kit (Invitrogen, California, USA), and the housekeeping gene GAPDH was used as the internal control. The SYBR Green assays were performed in triplicate on a 7500 real-time instrument (Applied Biosystems, CA, USA). The primers to detect mRNA were: COX-2, 5′-TATGAGTGTGGGATTTGACCAG-3′ and 5′-TCAGCATTGTAAGTTGGTGGAC-3′; CHOP, 5′-ACCACTCTTGACCCTGCTTCT-3′ and 5′ACTCTGTTTCCGTTTCCTGGT-3′; and GAPDH were 5′-AGAAGGCTGGGGCTCATTTG-3′ and 5′-AGGGGCCATCCACAGTCTTC-3′. All samples were normalized to internal controls and fold changes were calculated through relative quantification (2^−ΔΔCt^).

### Statistical Analysis

Three or more independent experiments were performed for each assay. Statistical analysis was performed by a Students’ t-test or ANOVA. Data are presented as means ± standard deviation (S.D.). Significance was noted at *P*<0.05.

## Results

### Induction of ER Stress Protects Hepatocellular Carcinoma Cells Against Apoptosis Induced by Doxorubicin

**Figure 1:**
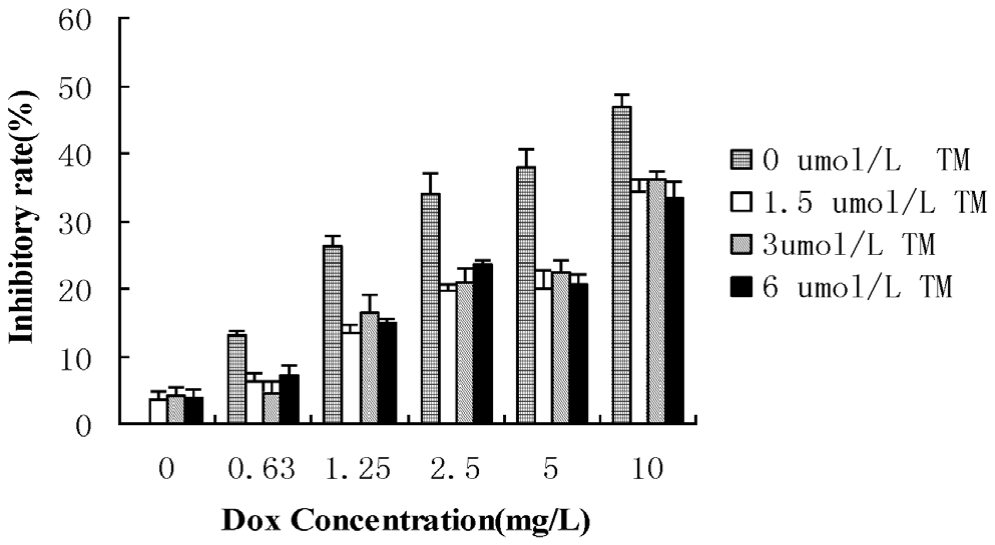
Effect of tunicamycin on cell viability induced by doxorubicin in HepG2 cells. HepG2 cells were pretreated with tunicamycin (0,1.5,3 and 6 µ mol/L) for 8 hr then exposed to different concentrations of doxorubicin (0, 0.63, 1.25, 2.5, 5 and 10 mg/L) for 24 hr. Cell viability of HepG2 cells was determined by the MTT assay. Data are expressed as the mean ± SD of three independent experiments (bars represent S.D.). (*P<0.05, **P<0.01, compared with HepG2 cells treated with doxorubicin alone).

**Figure 2:**
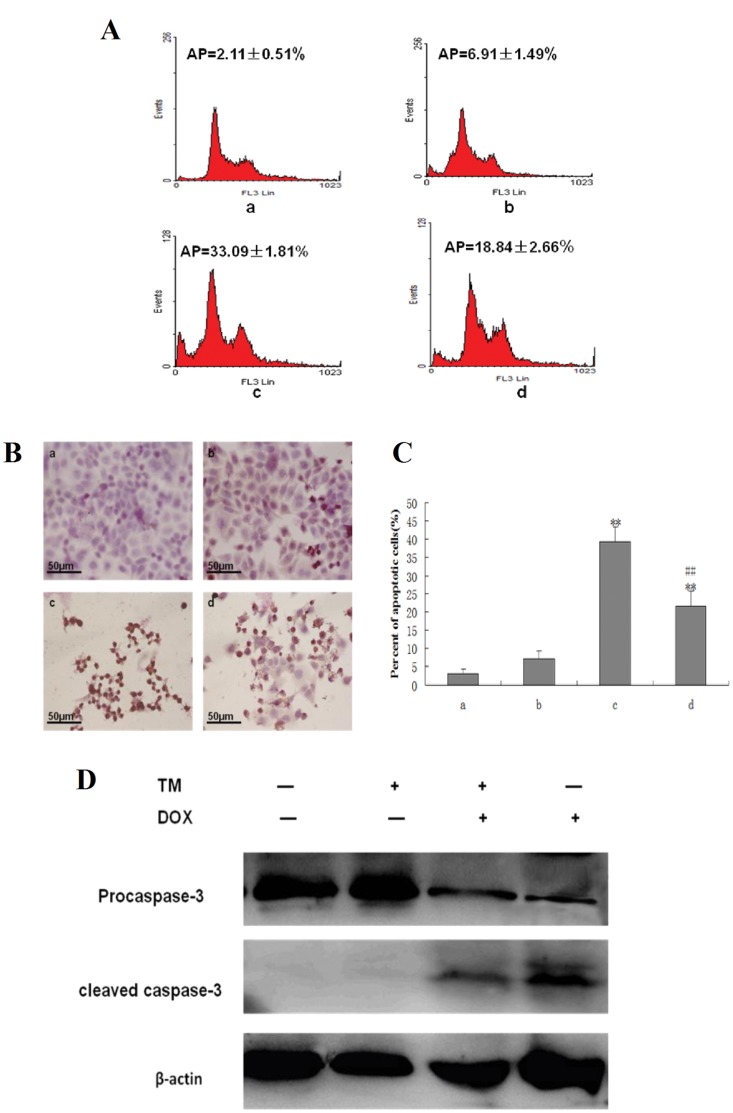
Effect of tunicamycin treatment on cell apoptosis induced by doxorubicin in HepG2 cells. (A) HepG2 cells were pretreated with 3 µmol/L tunicamycin (TM) for 8 hr and then exposed to doxorubicin (2.5 mg/L) for 24 hr. Sub-G1 analysis in HepG2 cells were determined by FACS, and the data are expressed as the mean ± SD of three independent experiments. a: Untreated HepG2 cells; b: HepG2 cells treated with tunicamycin alone; c: HepG2 cells treated with doxorubicin alone; d: HepG2 cells were pretreated with tunicamycin for 8 hr, and then exposed to doxorubicin for 24 hr. (B) and (C) Cell morphology and percentage of apoptotic cells was examined by TUNEL staining. In this representative image, the cells with brown nuclei are apoptotic cells. a: Untreated HepG2 cells; b: HepG2 cells treated with tunicamycin alone; c: HepG2 cells treated with doxorubicin alone; d: HepG2 cells were pretreated with tunicamycin for 8 hr, and then exposed to doxorubicin for 24 hr. Data are presented as mean ± SD of three independent experiments (bars represent S.D.). (**P<0.01, compared with untreated HepG2 cells; ^##^P<0.01, compared with doxorubicin alone) (D) Cleaved caspase-3 as an apoptotic marker were measured by western blot using specific anti- caspase-3 antibody. β-actin in the same HepG2 cells extract was used as an internal reference.

To determine the potential effects of ER stress on the sensitivity of hepatocellular carcinoma cells to chemotherapeutic agents, HepG2 cells were pretreated with the ER stress inducer tunicamycin (TM) for 8 hr before the addition of doxorubicin for 24 hr. Cell viability was assessed using the MTT assay (Figure 1). Pretreatment with different concentrations of TM significantly decreased doxorubicin-induced cytotoxicity in HepG2 cells between 0.63 to 10 mg/L of doxorubicin. Sub-G1 analysis was conducted by fluorescence-activated cell sorting analysis (FACS) and morphological changes, indicative of apoptosis, were also assessed by TUNEL staining. Consistent with the results of the MTT assay, treatment of HepG2 cells with doxorubicin (2.5 mg/L) resulted in a marked increase in the sub-G1 cell population (33.09%), which was significantly reduced (18.84%) in the presence of tunicamycin (Figure 2A). Similar results were observed through TUNEL staining (Figure 2B and2 C). Apoptosis was also measured through western blot analysis of cleaved caspase 3. As shown in Figure 2D, There was significantly lower levels of cleaved caspase 3 in HepG2 cells pretreated with tunicamycin compared to HepG2 cells treated with doxorubicin alone.

### Pae Reverses the Effects of ER Stress–induced Resistance to Doxorubicin

**Figure 3:**
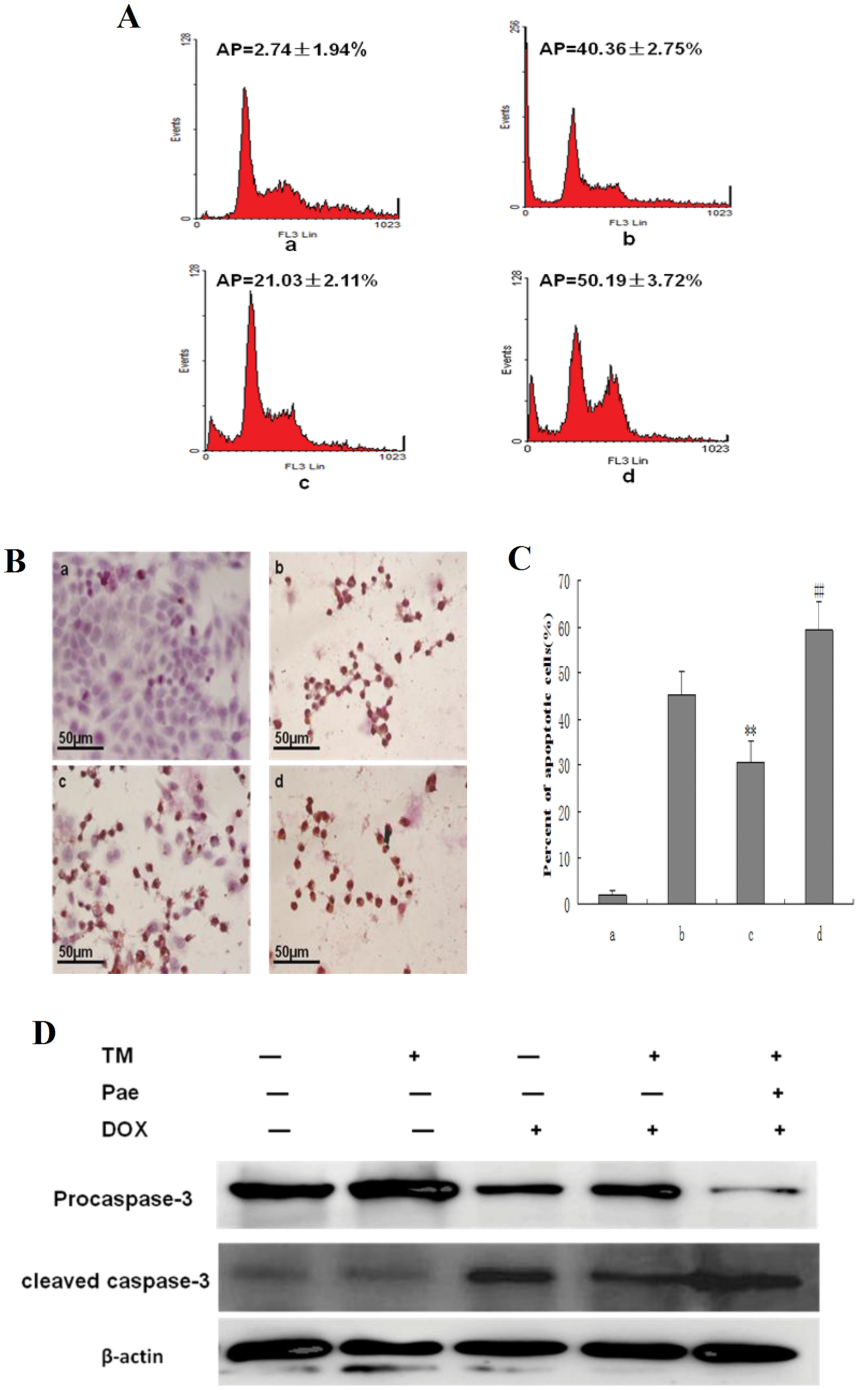
Effect of co-pretreatment with Pae and tunicamycin on apoptosis induced by doxorubicin in HepG2 cells. (A) HepG2 cells were treated with 3 µmol/L tunicamycin for 8 hr, either in the absence or the presence of 31.25 mg/L Pae and then exposed to doxorubicin (2.5 mg/L) for 24 hr. Apoptosis was analyzed as the sub-G1 fraction by fluorescence-activated cell sorting (FACS). a: Untreated HepG2 cells; b: HepG2 cells treated with doxorubicin alone; c: HepG2 cells co-pretreated with tunicamycin, and then exposed to doxorubicin for 24 hr d:HepG2 cells co-pretreated with tunicamycin and Pae, and then exposed to doxorubicin for 24 hr. (B) and(C) Cell morphology and percentage of apoptotic cells was examined by TUNEL staining. a: Untreated HepG2 cells; b: HepG2 cells treated with doxorubicin alone; c: HepG2 cells pretreated with tunicamycin, and then exposed to doxorubicin for 24 hr d:HepG2 cells co-pretreated with tunicamycin and Pae and then exposed to doxorubicin for 24 hr.Data are presented as mean ± SD for the three independent experiments. (**P<0.01 compared with HepG2 cells treated with doxorubicin alone, ^##^P<0.01 compared with HepG2 cells pretreated with tunicamycin, and then exposed to doxorubicin for 24 hr). (D) Cleaved caspase-3 as an apoptotic marker were measured by western blot using specific anti- caspase-3 antibody. β-actin in the same HepG2 cells extract was used as an internal reference.

To determine the effects of Pae on ER stress–induced resistance to doxorubicin, HepG2 cells were pretreated with Pae for 8 hr in the presence of tunicamycin, then subjected to doxorubicin treatment for 24 hr. Apoptosis was assessed by FACS analysis (Figure 3A), TUNEL staining (Figure 3B and 3C) and Western Blot (Figure 3D). Apoptosis induced by doxorubicin was increased by pretreatment of tunicamycin and Pae together. In the group treated with tunicamycin and Pae together the sub-G1 population increased sharply to 50.19% compared to cells pretreated with tunicamycin alone. Similar results were observed with TUNEL staining. In additon, When HepG2 cells were pretreated with tunicamycin and Pae combination treatment, significantly higher levels of cleaved caspase 3 were observed compared to HepG2 cells pretreated with tunicamycin.Together, these results support the inhibitory effect of Pae on ER stress–resistance to doxorubicin.

### Protection of Hepatocellular Carcinoma Cells against Doxorubicin-induced Apoptosis with Pretreatment of TM is Associated with COX-2

**Figure 4:**
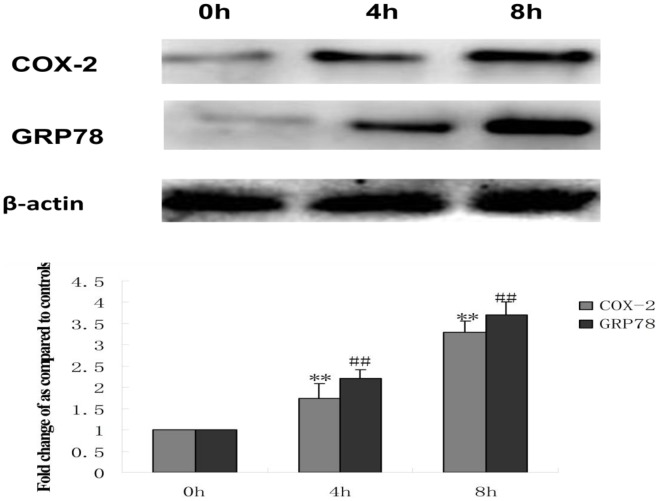
Effect of tunicamycin treatment on the expression of COX-2 and GRP78 in HepG2 cells. HepG2 cells were treated with 3 µmol/L tunicamycin (TM) for 0 (control), 4 and 8 hr. Equal protein amounts of cell lysates were subjected to western blot assay using specific anti-COX-2 and anti-GRP78 antibody. β-actin in the same HepG2 cells extract was used as an internal reference. Optical density reading values of the specific protein versus the loading control protein β-actin are represented as fold of the control values. (**P<0.01, ^##^P<0.01,compared with untreated HepG2 cells).

To investigate the underlying mechanisms involved in the protection of HepG2 cells treated with tunicamycin against doxorubicin-induced apoptosis, alterations in the protein expression of COX-2 and GRP78 (a hallmark of ER stress) were determined through western blotting. As shown in Figure 4, administration of tunicamycin to HepG2 cells induced an early increase in GRP78 expression, which is indicative of ER stress. Simultaneously, the expression of COX-2 rapidly increased after treatment with tunicamycin. These data suggest that COX-2 may be directly involved in ER stress–induced resistance to doxorubicin.

**Figure 5:**
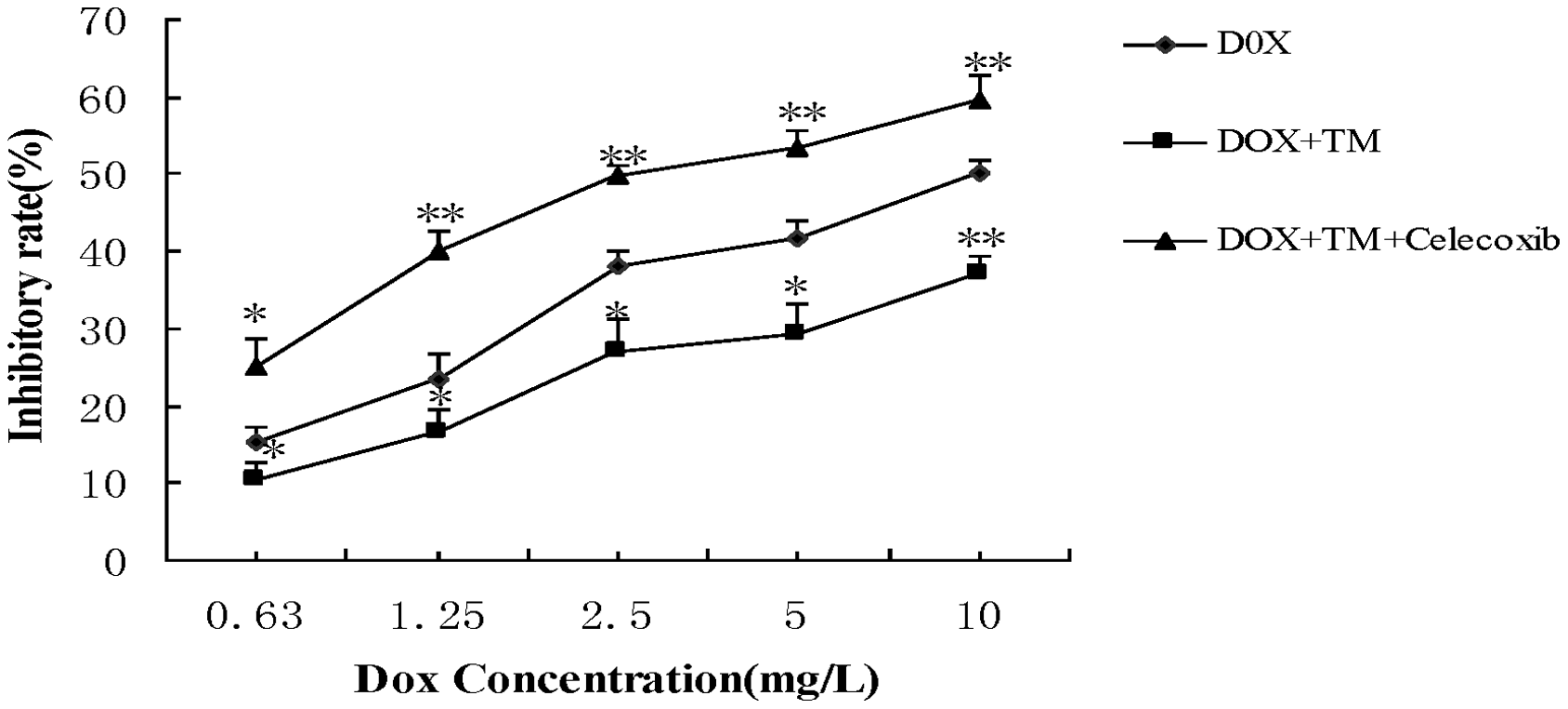
Effect of co-pretreatment with tunicamycin and celecoxib on cell viability induced by doxorubicin in HepG2 cells. HepG2 cells were pretreated with 3 µmol/L tunicamycin for 8 hr, either in the absence or the presence of celecoxib (50 µmol/L) and then exposed to doxorubicin (2.5 mg/L) for 24 hr. Cell viability of HepG2 cells was determined by the MTT assay. Data are expressed as the mean ± SD of three independent experiments (bars represent S.D.). (*P<0.05, **P<0.01, compared with HepG2 cells pretreated with tunicamycin, and then exposed to doxorubicin for 24 hr).

**Figure 6:**
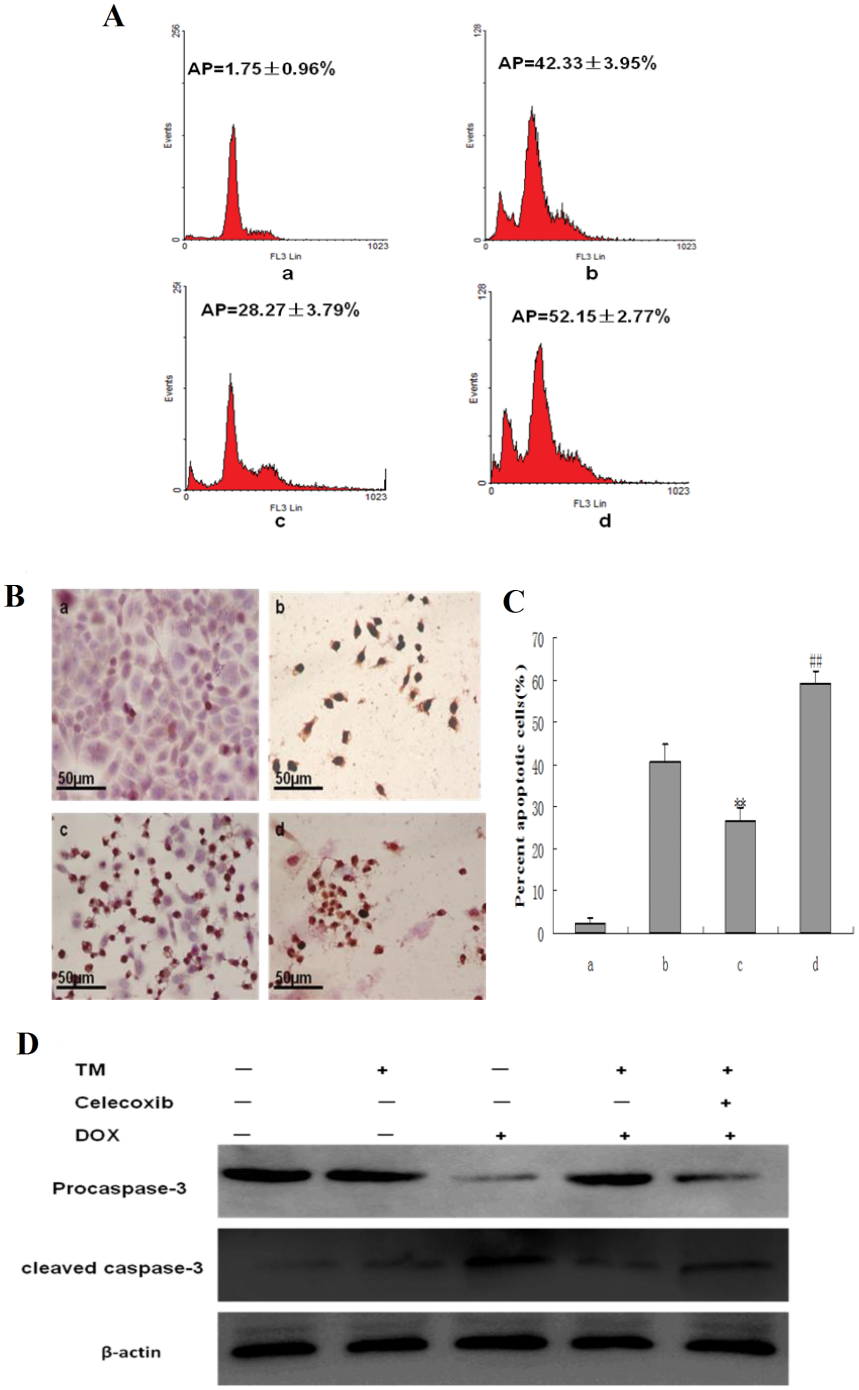
Effect of co-pretreatment with tunicamycin and celecoxib on apoptosis induced by doxorubicin in HepG2 cells. (A) HepG2 cells were pretreated with 3 µmol/L tunicamycin for 8 hr, either in the absence or the presence of celecoxib (50 µmol/L) and then exposed to doxorubicin (2.5 mg/L) for 24 hr. Apoptosis was analyzed as the sub-G1 fraction by fluorescence-activated cell sorting (FACS). a: Untreated HepG2 cells; b: HepG2 cells treated with doxorubicin alone; c: HepG2 cells pretreated with tunicamycin, and then exposed to doxorubicin for 24 hr d:HepG2 cells co-pretreated with tunicamycin and celecoxib, and then exposed to doxorubicin for 24 hr. (B) and(C) Cell morphology and percentage of apoptotic cells was examined by TUNEL staining. a: Untreated HepG2 cells; b: HepG2 cells treated with doxorubicin alone; c: HepG2 cells pretreated with tunicamycin, and then exposed to doxorubicin for 24 hr d: HepG2 cells co-pretreated with tunicamycin and celecoxib, and then exposed to doxorubicin for 24 hr. Data are presented as mean ± SD for the three independent experiments. (**P<0.01 compared with HepG2 cells treated with doxorubicin alone,^ ##^P<0.01 compared with HepG2 cells pretreated with tunicamycin, and then exposed to doxorubicin for 24 hr). (D) Cleaved caspase-3 as an apoptotic marker were measured by western blot using specific anti- caspase-3 antibody. β-actin in the same HepG2 cells extract was used as an internal reference.

**Figure 7:**
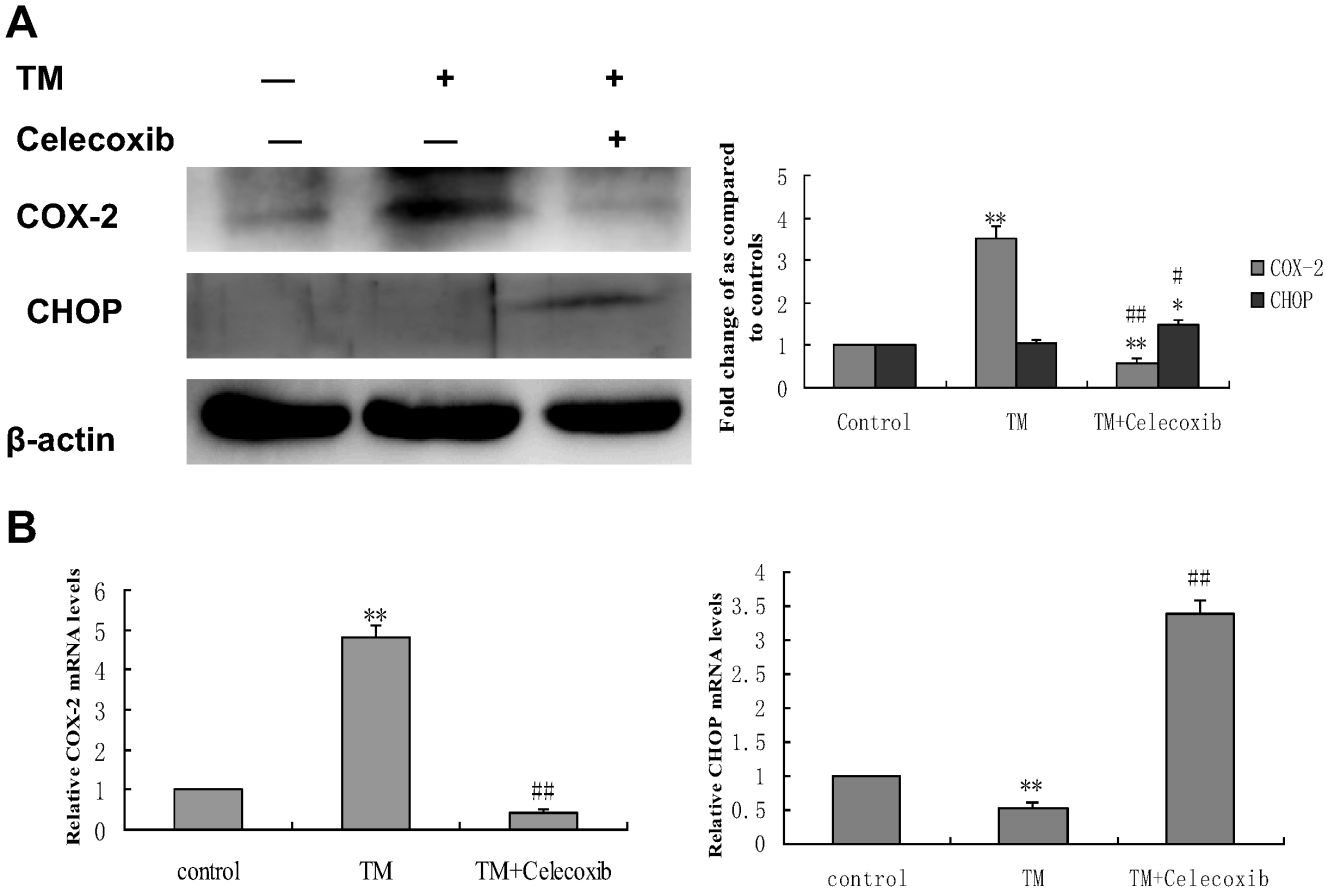
Effect of co-treatment with tunicamycin and celecoxib on the expression of COX-2 and CHOP in HepG2 cells. HepG2 cells were treated with 3 µmol/L tunicamycin in either the absence (control) or the presence of celecoxib (50 µmol/L) for 8 hr. (A)Equal amounts of cell lysates were subjected to western blot analysis using specific anti-COX-2 and anti-CHOP antibody. β-actin in the same HepG2 cells extract was used as an internal used as an internal reference. Optical density reading values of the specific protein versus the loading control protein β-actin are represented as fold of the control values. (^*^P<0.05, ^**^P<0.01, compared with untreated HepG2 cells, ^#^P<0.05, ^##^P<0.01, compared with HepG2 cells treated with tunicamycin alone). (B) RNA was harvested and gene expression examined by qRT-PCR. The qRT-PCR fold-changes were normalised using the expression of a housekeeping gene (GAPDH) and compared with those obtained from untreated HepG2 cells.Data are presented as mean ± SD for the three independent experiments. (**P<0.01 compared with untreated HepG2 cells,^ ##^P<0.01 compared with HepG2 cells pretreated with tunicamycin).

To confirm that COX-2, which was activated by ER stress, indeed enhances doxorubicin–induced apoptosis, HepG2 cells were pretreated with tunicamycin in the presence of celecoxib, a selective COX-2 inhibitor, for 8 hr, followed by doxorubicin treatment for 24 hr. Inhibition of cell growth was determined by the MTT assay. Celecoxib significantly reduced cell proliferation when co-pretreated with tunicamycin (Figure 5). Apoptosis was also quantified by FACS analysis (Figure 6A) and TUNEL staining (Figure 6B and 6C) and Western-blot(Figure 6D). Co-pretreatment of both celecoxib and tunicamycin significantly increased the percentage of sub-G1 cells and the number of TUNEL-positive HCC cells as well as the levels of cleaved caspase 3. CHOP (CCAAT/enhancer-binding protein homologous protein), also called GADD153, is one of the primary effectors of ER stress–mediated cell apoptosis [20]. Western blotting and qRT-PCR analysis was also performed to detect the expression of CHOP. As shown in Figure 7A and 7B, the protein expression and the expression of CHOP mRNA was markedly increased in the presence of celecoxib and tunicamycin. These data indicate that down-regulation of COX-2 could inhibit the effect of tunicamycin on doxorubicin-induced apoptosis.

### PI3K/AKT/CHOP Pathway is Implicated in the COX-2 Mediated Cytoprotective Function of ER Stress against Doxorubicin-induced Hepatocellular Carcinoma Cells Apoptosis

**Figure 8:**
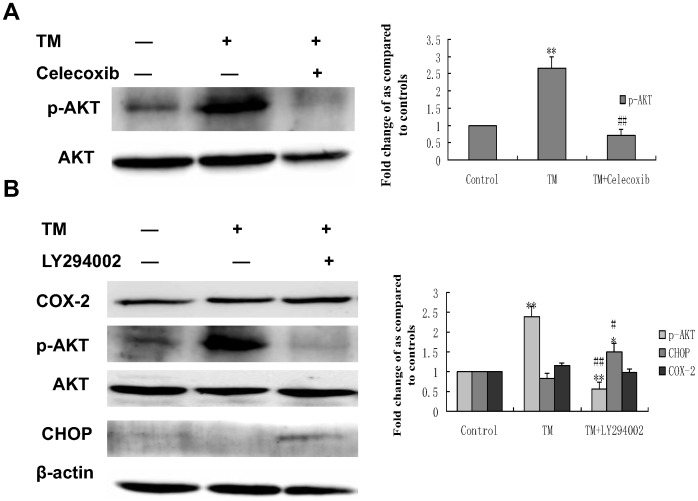
Effect of co-treatment with tunicamycin and celecoxib/LY294002 on the expression of phospho (p)-Akt and CHOP in HepG2 cells. HepG2 cells were treated with 3 µmol/L tunicamycin in either the absence (control) or the presence of celecoxib(A)(50 µmol/L)/LY294002(B) (30 µmol/L) for 8 hr. Equal amounts of cell lysates were subjected to western blot analysis using specific anti-COX-2, anti-p-AKT, anti-AKT and anti-CHOP antibody. β-actin in the same HepG2 cells extract was used as an internal used as an internal reference. Optical density reading values of the specific protein versus the loading control protein β-actin are represented as fold of the control values. (*P<0.05, **P<0.01, compared with untreated HepG2 cells, ^#^P<0.05, ^##^P<0.01, compared with HepG2 cells treated with tunicamycin alone).

To elucidate which signaling pathway is involved in COX-2 mediated cytoprotective function of ER stress, the activation status of the PI3K/Akt survival pathway was monitored in HepG2 cells before and after treatment with celecoxib in the presence of tunicamycin. As shown in Figure 8A, exposure to TM increases activation (phosphorylation) of Akt, whereas celecoxib inhibited TM-induced activation of Akt. Furthermore, HepG2 cells treated with the PI3K inhibitor, LY294002, in the presence of TM increased the expression of CHOP protein. (Figure 8B), These results suggest that the PI3K/AKT/CHOP pathway is important in the COX-2 mediated cytoprotective function of ER stress against doxorubicin-induced hepatocellular carcinoma cells apoptosis.

### COX-2 Mediated Inactivation of the PI3K/AKT Pathway is Involved in the Reversing Effect of Pae on Endoplasmic Reticulum Stress–induced Resistance to Doxorubicin in Human Hepatocellular Carcinoma Cells

**Figure 9:**
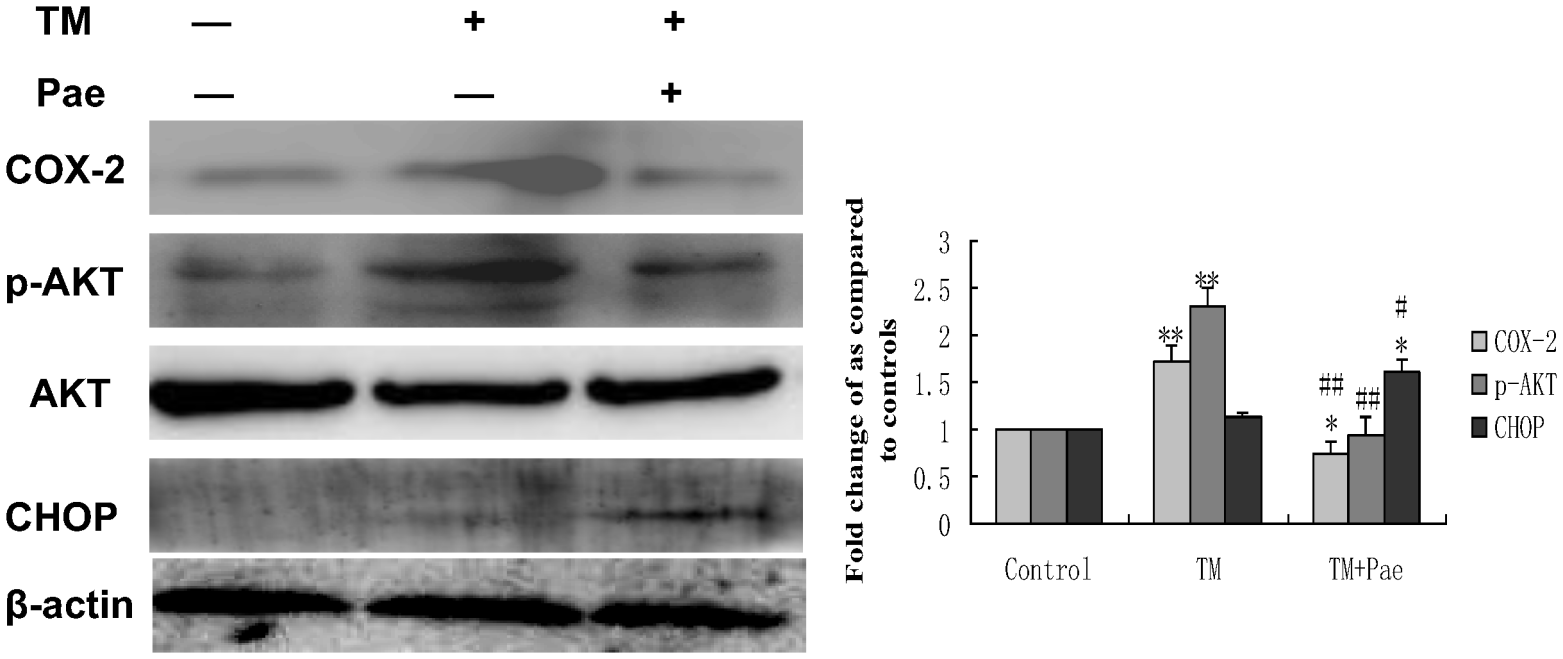
Effect of co-treatment with Pae and tunicamycin on the expression of COX-2, phospho (p)-Akt and CHOP in HepG2 cells. Whole cell lysates from HepG2 cells with treatment with 3 µmol/L tunicamycin (TM) in either the absence (control) or the presence of 31.25 mg/LPae for 8 hr were subjected to western blotting analysis. β-actin in the same HepG2 cells extract was used as an internal used as an internal reference. Optical density reading values of the specific protein versus the loading control protein β-actin are represented as fold of the control values. (*P<0.05, **P<0.01, compared with untreated HepG2 cells, ^#^P<0.05, ^##^P<0.01, compared with HepG2 cells treated with tunicamycin alone).

**Figure 10:**
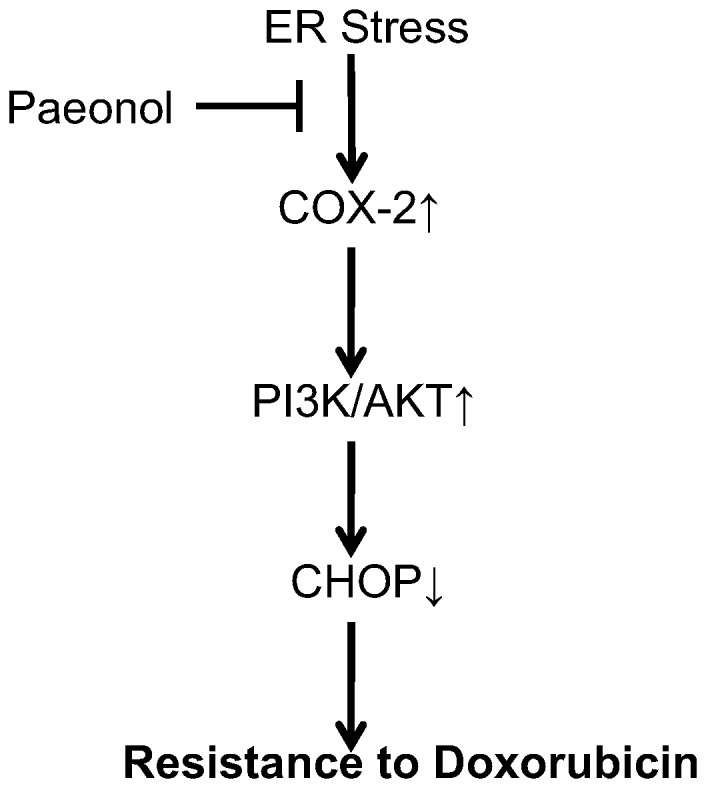
Schematic model of ER stress-mediated resistance to doxorubicin reversed by Paeonol in human hepatocellular carcinoma cells.

Western blot analysis was conducted to determine whether COX-2 induced by tunicamycin could be altered by Pae treatment. Pae significantly decreased the levels of COX-2 induced by tunicamycin (Figure 9). In addition, activation of Akt decreased and the levels of CHOP were increased with paeonol treatment (Figure 9). These results suggest that Pae may have an inhibitory effect on ER stress–resistance to doxorubicin by targeting the PI3K/AKT/CHOP pathway via COX-2 (Figure.10).

## Discussion

Reduced chemosensitivity of solid cancer cells represents a pivotal obstacle in clinical oncology. The uncontrolled growth and insufficient vascularization of a tumor mass leads to a stressed state in the tumor microenvironment, which includes low oxygen supply, nutrient deprivation and pH changes [10]–[11]. Many intrinsic pathophysiologic conditions, such as hypoxia, anoxia and glucose deprivation may account for tumor resistance to chemotherapeutic drugs [12]–[13]. But how this contributes to chemoresistance in HCC cells remains to be explored. Evidence is emerging that microenvironment stresses directly impinge on the luminal milieu of the endoplasmic reticulum (ER) and may cause ER stress [14]. These ER stress conditions activate a range of cellular stress response pathways, including inappropriate activation of survival or proliferative pathways [7]–[9]. Therefore, we hypothesized that a stressful environmental milieu in the ER might induce intrinsic resistance to chemotherapeutic drugs. In order to test this concept, we first established an ER stress microenvironment in HepG2 cells by use of exogenous stimulus. Based on this mimicked ER stress microenvironment, we investigated whether the ER stress condition plays a role in regulating doxorubicin-mediated HepG2 cell cytotoxicity and apoptosis through the MTT assay, FACS analysis, TUNEL staining and Western-blot. Pretreatment with TM for 8 hr decreased the levels of cytotoxicity induced by doxorubicin in HepG2 cells. Furthermore, treatment of HepG2 cells with the anticancer drug doxorubicin resulted in an increase in the sub-G1 cell population (33.09%), but this population was significantly reduced (18.84%) in the presence of tunicamycin.The numbers of fragmented nuclei in the doxorubicin-treated HepG2 cells were also reduced in the presence of tunicamycin. Consistent with the FACS analysis data and TUNEL staining, the expression of cleaved caspase 3,as an apoptotic marker, was decreased in the presence of tunicamycin. Our data indicates that tunicamycin pre-incubation significantly reduced doxorubicin–induced apoptosis in HCC cells. The evidence supports the observation that ER stress may induce resistance to doxorubicin.

Given the observation that ER stress is correlated with drug resistance, agents that restore ER-mediated drug resistance could define a new strategy of cancer therapy. Pae is isolated from the herb Pycnostelma paniculatum (Bunge) K.S., and the root of the plant Paeonia Suffruticosa Andrew [15]. Pae is a white needle crystal with a relatively low melting point of 51°C–52°C. The molecular weight of Pae is 166.18 kD and the molecular formula is C_9_H_10_O_3_. Pae has minimal systemic toxicity (LD50 3430 mg/kg) when orally administrated to mice [21]. In our previous study, the anti-neoplastic activity of Pae was demonstrated both in cell lines, such as human erythromyeloid cell line (K562), breast cancer gene cell line (T6-17), human hepatoma cell line (Bel-7404), and a cervical cancer cell line (Hela), and in animal models bearing HepA hepatocarcinoma [22]–[23]. Recent studies have shown that Pae is a potent therapeutic agent in combination with other drugs to support the efficacy of conventional anticancer agents and also to reduce their side effects [24]–[25]. Nevertheless, the effect of Pae on ER stress–induced resistance to doxorubicin has not been reported. Interestingly, our data showed that apoptosis induced by doxorubicin in HepG2 cells was increased by co-pretreatment of tunicamycin and Pae. The sub-G1 percentage and the number of apoptotic cells stained through TUNEL in both Pae and tunicamycin-pretreated HepG2 cells increased sharply up to 50.19%, compared to cells pretreated with tunicamycin.The expression of cleaved caspase 3, was also increased in the presence of tunicamycin and Pae. In the presence of Pae, protection against doxorubicin-induced apoptosis with pretreatment of TM was abrogated.

In order to explore the precise mechanisms responsible for the reversing effect of Pae on doxorubicin-induced apoptosis in human hepatocellular carcinoma cells, we first investigated the mechanism underlying doxorubicin resistance. COX-2 is the inducible form of cyclooxygenase, which is frequently elevated in cancer tissues. Therefore, COX-2 has been suggested to play a major role in tumorigenesis [26]–[27]. Recent studies have reported that COX-2 regulates multiple cellular processes including survival, proliferation, and apoptosis in cancer [28]–[29]. Upon ER stress, COX-2 is strongly activated in the mouse liver immortalized cell line ML-1 or human breast cancer cell line MCF-7 [30]. These reports suggest that the COX-2 is closely associated with ER stress induction. In view of this evidence, we considered whether the cytoprotective responses induced by ER stress in hepatoma cells can be attributed to COX-2. If COX-2 expression is protective, then targeting COX-2 may reverses the sensitivity of hepatoma cells to ER stress–induced apoptosis. We examined the COX-2 status of hepatoma cells during ER stress. In the search for the protective mechanism(s) against doxorubicin resulting from preloaded ER stress, we found that exposure to TM increased the expression of COX-2. TM induced COX-2 expression as early as 4 hours. Moreover, when the COX-2 inhibitor celecoxib was used, its effect on TM-mediated protection against doxorubicin was attenuated and the levels of CHOP increased. Taken together, these results indicate that the inhibition of COX-2 sensitizes human hepatocellular carcinoma cells to doxorubicin-induced apoptosis. To pursue the potential relationship between COX-2 and Akt phosphorylation in hepatocellular carcinoma cells, we next questioned whether COX-2 is responsible for Akt phosphorylaiton or is in the reverse. Inhibiton of COX-2 expression using celecoxib was enough to abrogate the phosphorylation of Akt and increase the expression of CHOP. On the contrary, inhibition of Akt phosphorylation by PI3K inhibitor, LY294002, did not affect COX-2 levels, but increased the levels of CHOP. These data clearly demonstrate that Akt is a downstream target of COX-2 in hepatocellular carcinoma cells. PI3K/AKT pathway is implicated in COX-2 mediated cytoprotective function of ER stress in doxorubicin-induced HCC cell apoptosis. Moreover, we also showed that Pae reduced the expression of COX-2 and subsequently downregulated phosphorylation of AKT in HepG2 cells. This effect was similar using the COX-2 inhibitor, celecoxib. Since Pae also had a similar reversing effect on in the same cells, it suggests that Pae exerts its reversing activity, possibly via COX-2 mediated-PI3K/AKT/CHOP pathway. (Figure 10).

In conclusion, our studies are the first to reveal evidence that Pae reverses the cellular effects of ER stress–induced resistance to doxorubicin in hepatocellular carcinoma cells. These findings provide important new insights that Pae may be a promising approach in abrogating ER stress–induced resistance to chemotherapeutic agents as a therapeutic strategy for the treatment of HCC and other cancers. Presently, it is unknown whether the reversing effects of Pae in ER stress–induced resistance to doxorubicin observed *in vitro* will also work *in vivo*. A number of clinical and basic studies are required to establish the effects of Pae on specific cancer types.
